# Fibrinogen E fragment selectively disrupts the vasculature and inhibits the growth of tumours in a syngeneic murine model

**DOI:** 10.1038/sj.bjc.6600320

**Published:** 2002-06-05

**Authors:** N J Brown, C A Staton, G R Rodgers, K P Corke, J C E Underwood, C E Lewis

**Affiliations:** Microcirculation Research Unit, University of Sheffield Medical School, Sheffield S10 2RX, UK; Tumour Targeting Group, University of Sheffield Medical School, Sheffield S10 2RX, UK; Institute for Cancer Studies, University of Sheffield Medical School, Sheffield S10 2RX, UK

**Keywords:** angiogenesis, anti-angiogenesis, tumour growth, endothelial cells, thrombosis

## Abstract

We recently demonstrated that a fragment of human fibrinogen, fibrinogen E fragment, inhibits the migration and differentiation of human endothelial cells *in vitro*. Here we show that it exerts similar effects on murine endothelial cells *in vitro*, and selectively disrupts tumour endothelium *in vivo*, causing widespread intravascular thrombosis and retarding the growth of CT26 tumours in a syngeneic murine model.

*British Journal of Cancer* (2002) **86**, 1813–1816. doi:10.1038/sj.bjc.6600320 www.bjcancer.com

© 2002 Cancer Research UK

## 

Angiogenesis, the growth of new capillaries from an existing host vascular bed, involves the migration, proliferation and differentiation of endothelial cells and is crucial for the growth and metastasis of tumours (Folkman, 1995). Various haemostatic proteins regulate tumour angiogenesis (Browder *et al*, 2000), including a 50 kDa proteolytic fragment (fibrinogen E fragment, FgnE) of the blood clotting protein, fibrinogen, which inhibits human endothelial cell activity *in vitro* (Bootle-Wilbraham *et al*, 2000).

Here we first established that FgnE also inhibits the activity of *murine* human endothelial cells *in vitro*, and then examined the effects of daily injections of FgnE on the vascular status and growth of CT26 tumours in Balb/C mice *in vivo* (a syngeneic tumour model).

## MATERIALS AND METHODS

### Cells and cell culture

The murine colonic adenocarcinoma cell line, CT26, is a N-nitroso-N-methylurethane induced Balb/C tumour and was a gift from Professor Ian Hart, ICRF, London, UK. An SV40-transformed murine endothelial cell line, SVEC4-10, was obtained from ATCC. Both cell types were maintained in Dulbecco's Minimal Eagles Medium (DMEM), 10% foetal calf serum (FCS), 1% penicillin and streptomycin in 5% CO_2_ in air at 37°C.

### Proteins and peptides

Human FgnE was purchased from Diagnostica Stago (Asnieres, France). Recombinant human VEGF_165_ was purchased from R&D Systems (Abingdon, UK).

### *In vitro* assays

The MTT proliferation assay was used as described previously (Liu *et al*, 1999) following the incubation of SVEC4-10 in DMEM+10% FCS in the presence of 0, 10 or 100 nM FgnE for 24, 48 and 96 h. The migration assay involved use of a microchemotaxis chamber (Neuro Probe Inc, Cabin John, MD, USA) with 8 μm pore size filter coated with collagen type IV (Bootle-Wilbraham *et al*, 2000). Ten ng ml^−1^ VEGF with or without 10 or 100 nM FgnE were placed in the lower wells. SVEC4-10 cells in DMEM containing 1% FCS were added to the upper chamber for 4.5 h and then migrated cells on the bottom of the filters fixed, stained and counted at ×160 magnification in three random fields per filter. The Matrigel assay was performed as described in Donovan *et al* (2001). SVEC4-10 cells were seeded onto wells coated with growth factor-reduced (GF-reduced) Matrigel (Becton Dickinson Labware, Bedford, MA, USA) and incubated for 6 h in DMEM+1% FCS alone (control), or medium±10 ng ml^−1^ VEGF±10 or 100 nM FgnE. Cells were then fixed, stained with haematoxylin and eosin, visualized (at ×40 magnification), and tubule formation assessed in three randomly selected fields of view per well using Scion Image software. Cell viability was assessed by seeding SVEC4-10 cells in the absence or presence of 0, 10 or 100 nM FgnE for 24 h (Bootle-Wilbraham *et al*, 2002) and adding propidium iodide prior to FACScanning using Cell Quest software (Becton Dickinson).

### *In vivo* study

All experiments were performed under HO Project Licence Number PPL40/1557 (to NJ Brown) and conformed to the United Kingdom Co-ordinating Committee on Cancer Research (UKCCCR) Guidelines for the Welfare of Animals in Experimental Neoplasia (as described in 1998 in *Br J Cancer* **77:** 1–10). Animals were anaesthetised by intraperitoneal injection of 1 : 1 diazepam and hypnorm (Janssen, UK). Mice were then inoculated subcutaneously with 2×10^6^ ml^−1^ in 100 μl viable CT26 cells. When tumours had grown to 100–350 mm^3^, mice were injected i.p. daily for 8 days with FgnE (0.5 mg kg^−1^; eight mice) or vehicle (phosphate buffered saline; seven mice). Assuming 50–100% absorption from the peritoneum into the bloodstream, and a total blood volume in mice of 2 ml, an injection of 0.5 mg kg^−1^ FgnE was calculated to result in a plasma concentration of 10–100 nM (i.e. similar to the concentration used in our *in vitro* studies). Tumour volumes were calculated daily using calipers (Grosios *et al*, 1999). After 8 days, mice were killed and tumours and normal tissues excised, divided into two halves and fixed in either: (i) 10% neutral buffered formalin or (ii) zinc-based fixative (Beckstead, 1994), the processed into paraffin wax.

Formalin-fixed sections were stained for H&E or Martius yellow-brilliant crystal scarlet-soluble blue (MSB, a histological stain for fibrin) (Lendrum *et al*, 1962). Using consecutive microscopic fields, tumour necrosis was assessed semi-quantitatively using a Chalkley grid method (% necrosis). Zinc-fixed sections were exposed to a rat monoclonal anti-murine CD31 (1 : 100; Pharminogen, CA, USA) specific for endothelial cells, for 60 min at room temperature, and immunoreactivity detected using the ABC rat elite kit (Vector Laboratories, UK) and diaminobenzidine. Maximal vascular density was quantified using the Chalkley grid method (Leek *et al*, 1996).

### Statistical analysis

**Figure 1:**
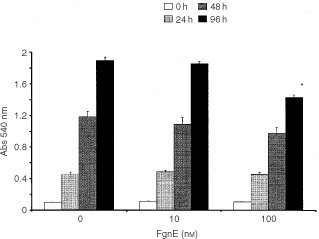
Effects of FgnE (10 and 100 nM) on the proliferation of SVEC4-10 cells *in vitro*. *P<0.05 w.r.t. the same time point for the relevant control group.

**Figure 2:**
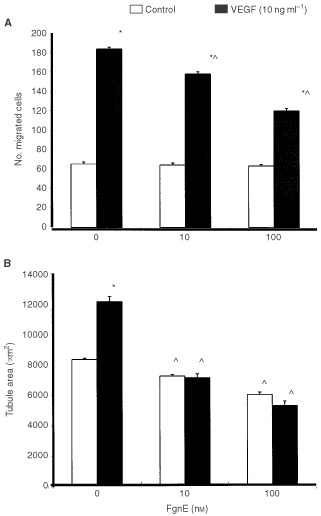
Inhibitory effects of FgnE on the (**A**) migration of, or (**B**) tubule formation by, SVEC4-10 cells on growth factor-reduced Matrigel *in vitro* (in the absence (control) or presence of 10 ng ml^−1^ exogenous recombinant VEGF). *P<0.005 w.r.t. respective ‘no VEGF’ group, ^*P*<0.01 w.r.t. respective ‘no FgnE’ group.

**Figure 3:**
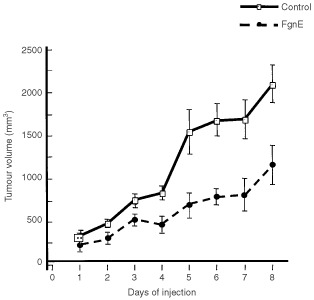
FgnE inhibition of the growth of CT26 tumours in Balb/C mice. Mice were injected i.p. with PBS alone (control) or 0.5 mg kg^−1^ FgnE in PBS for 8 days. Tumour volume was significantly (*P*<0.05) reduced in FgnE-treated animals from day 2 of the injection period onwards.

Statistical analysis was performed using the Mann–Whitney *U*-test. All data shown in Figures 1–3

**Figure 4:**
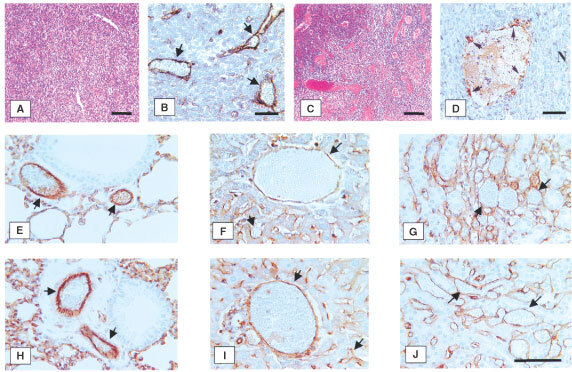
Effect of FgnE injection on the general histology and vascular endothelium of malignant and non-malignant murine tissues *in vivo*. CT26 tumours were excised from control (**A**,**B**) or FgnE-treated (**C**,**D**) mice and stained with haematoxlin and eosin, and morphology examined at low magnification (**A**,**C**; bars=100 μm) or anti-murine CD31 (brown stain; see arrows) and viewed at higher magnification (**B**,**D**; bars=50 μm). Cells in control tumours exhibited a compact, regular morphology (**A**) with vessels lined by a continuous single layer of endothelial cells (**B**). By contrast, FgnE-treated tumours exhibited an increased levels of necrosis (see ‘N’ in **D**) and large distended vessels (as in **C**,**D**) lined with discontinuous endothelial cells (see arrows in **D**). CD31-positive cells lining vessels in a range of non-malignant tissues (see arrows); lungs (**E**,**H**), lever (**F**,**I**) and kidneys (**G**,**J**), from mice were unaffected by FgnE treatment (**H**,**I** and **J**) and resembled those from control (**E**,**F** and **G**) mice. Bar in **J**=50 μm (same magnification in **E**–**J**).

Figure 2

Figure 3

are means±s.e.m. and representative data from one of three replicate experiments are shown.

## RESULTS

### *In vitro* assays

One hundred nM FgnE significantly (*P*<0.05) inhibited the proliferation of SVEC4-10 cells over 96 h *in vitro* (Figure 1). VEGF-induced migration of SVEC4-10 across collagen-coated filters was significantly (*P*<0.01) inhibited by exposure to both 10 or 100 nM FgnE (Figure 2A). SVEC4-10 cells formed tubules in the absence of VEGF, but this was significantly (*P*<0.001) enhanced by VEGF. Addition of FgnE (10 or 100 nM) also significantly (*P*<0.005) inhibited VEGF-stimulated tubule formation (Figure 2B). Neither dose of FgnE was cytotoxic for SVEC4-10 cells.

### *In vivo* studies

Control tumours grew steadily over the 8-day injection period but FgnE injections significantly (*P*<0.001) retarded the growth of tumours from day 2 onwards (Figure 3). FgnE injections were well tolerated *in vivo* with no significant effect on body weight or the general well-being of the animals. A central area of necrosis consisting of cell debris alone was evident in both control and FgnE-treated tumours, but was significantly (*P*<0.05) larger in FgnE-treated mice (% necrosis: 7.8±0.9 for control *versus* 23.9±2.8 for FgnE-treated). No significant difference in microvessel counts was seen, although this was slightly higher in areas abutting the central necrotic areas in FgnE-treated (6.5±0.9) than control (4.8±0.5) tumours, because these vessels were distended, lined with patchy, incomplete endothelial cells and filled with/or surrounded by fibrin (Figure 3D) as seen by MSB staining (indicating that thrombus formation had occurred in these damaged vessels). However, FgnE had no effect on the endothelium in the normal tissues examined (lungs, liver and kidneys). Similar results were obtained in three separate cohorts of animals.

## DISCUSSION

This study is the first to show that FgnE inhibits the proliferation, migration and tubule formation of *murine* endothelial cells *in vitro*, and causes widespread damage to blood vessels in CT26 tumours *in vivo*. The resultant microvascular thrombosis and occlusion in tumour blood vessels caused widespread destruction of tumour cells and retarded tumour growth. The selective disruption of tumour endothelium by FgnE indicates that this fragment may bind with greater affinity to activated tumour endothelium than quiescent endothelial cells in healthy tissues. However, further studies are needed to show whether the inhibitory effects of FgnE on endothelial cells *in vitro* contributed significantly to the anti-vascular effects seen *in vivo*. It would also be interesting to determine whether higher doses of FgnE, or administration of FgnE by a different route (e.g. intravenous) would enhance this anti-tumour effect.

The receptor(s) mediating the inhibitory effects of FgnE on endothelial cells has/have yet to be identified. Previous studies have shown that whole fibrinogen binds to various integrins on endothelial cells via RGD motifs in the D-domain of the fibrinogen molecule (Suehiro *et al*, 1997). However, FgnE lacks these domains, so FgnE binding may involve one or more novel, non-RGD sites. Interestingly, FgnE (100 nM) also significantly inhibited the proliferation (but not the migration or viability) of CT26 cells and 3T3 fibroblasts over 96 h in our assays, suggesting that it may have a direct effect on tumour cells *in vivo* (data not shown).

Taken together, these preliminary findings suggest that FgnE could be used to selectively target blood vessels in tumours, causing endothelial cell damage, triggering a haemostatic response (i.e. thrombosis), leading to inadequate perfusion and widespread tumour cell death. This fragment may also exert a direct anti-proliferative effect on some types of tumour cell, thereby contributing to retarded tumour growth by this second mechanism.
